# Socio-economic determinants of physical activity across the life course: A "DEterminants of DIet and Physical ACtivity" (DEDIPAC) umbrella literature review

**DOI:** 10.1371/journal.pone.0190737

**Published:** 2018-01-19

**Authors:** Grainne O’Donoghue, Aileen Kennedy, Anna Puggina, Katina Aleksovska, Christoph Buck, Con Burns, Greet Cardon, Angela Carlin, Donatella Ciarapica, Marco Colotto, Giancarlo Condello, Tara Coppinger, Cristina Cortis, Sara D’Haese, Marieke De Craemer, Andrea Di Blasio, Sylvia Hansen, Licia Iacoviello, Johann Issartel, Pascal Izzicupo, Lina Jaeschke, Martina Kanning, Fiona Ling, Agnes Luzak, Giorgio Napolitano, Julie-Anne Nazare, Camille Perchoux, Caterina Pesce, Tobias Pischon, Angela Polito, Alessandra Sannella, Holger Schulz, Chantal Simon, Rhoda Sohun, Astrid Steinbrecher, Wolfgang Schlicht, Ciaran MacDonncha, Laura Capranica, Stefania Boccia

**Affiliations:** 1 School of Public Health, Physiotherapy & Sports Science, University College Dublin, Dublin, Ireland; 2 School of Health and Human Performance, Dublin City University, Dublin, Ireland; 3 Section of Hygiene—Institute of Public Health; Università Cattolica del Sacro Cuore, L.go F. Vito, Rome, Italy; 4 Leibniz Institute for Prevention Research and Epidemiology, BIPS, Bremen, Germany; 5 Dept of Sport, Leisure and Childhood Studies, Cork Institute of Technology, Cork, Ireland; 6 Department of Movement and Sports Sciences, Ghent University, Ghent, Belgium; 7 Department of Physical Education and Sports Sciences, University of Limerick, Limerick, Ireland; 8 Council for Agricultural Research and Economics -Research Centre for Food and Nutrition, Rome, Italy; 9 Department of Movement, Human and Health Sciences, University of Rome Foro Italico, P.za Lauro de Bosis, Rome, Italy; 10 Department of Human Sciences, Society and Health, University of Cassino and Lazio Meridionale, Cassino, Italy; 11 Department of Medicine and Aging Sciences, 'G. d'Annunzio' University of Chieti-Pescara, Chieti and Pescara, Italy; 12 Department for Sport and Exercise Sciences, University of Stuttgart, Stuttgart, Germany; 13 Department of Epidemiology and Prevention. IRCCS Instituto Neurologico Mediterraneo: NEUROMED. Pozzilli, Italy; 14 Molecular Epidemiology Group, Max Delbrueck Center for Molecular Medicine in the Helmholtz Association (MDC), Berlin, Germany; 15 Department of Sports Science, University of Konstanz, Konstanz, Germany; 16 Institute of Epidemiology I, Helmholtz Zentrum München, German Research Center for Environmental Health, Neuherberg, Germany; 17 CarMeN Laboratory, INSERM U1060, Lyon 1 University, CRNH-Rhône-Alpes, CENS, Lyon, France; University of Lausanne Hospital Centre, SWITZERLAND

## Abstract

**Background:**

To date, the scientific literature on socioeconomic correlates and determinants of physical activity behaviours has been dispersed throughout a number of systematic reviews, often focusing on one factor (e.g. education or parental income) in one specific age group (e.g. pre-school children or adults). The aim of this umbrella review is to provide a comprehensive and systematic overview of the scientific literature from previously conducted research by summarising and synthesising the importance and strength of the evidence related to socioeconomic correlates and determinants of PA behaviours across the life course.

**Methods:**

Medline, Embase, ISI Web of Science, Scopus and SPORTDiscus were searched for systematic literature reviews and meta-analyses of observational studies investigating the association between socioeconomic determinants of PA and PA itself (from January 2004 to September 2017). Data extraction evaluated the importance of determinants, strength of evidence, and methodological quality of the selected papers. The full protocol is available from PROSPERO (PROSPERO2014:CRD42015010616).

**Results:**

Nineteen reviews were included. Moderate methodological quality emerged. For adults, convincing evidence supports a relationship between PA and socioeconomic status (SES), especially in relation to leisure time (positive relationship) and occupational PA (negative relationship). Conversely, no association between PA and SES or parental SES was found for pre-school, school-aged children and adolescents.

**Conclusions:**

Available evidence on the socioeconomic determinants of PA behaviour across the life course is probable (shows fairly consistent associations) at best. While some evidence is available for adults, less was available for youth. This is mainly due to a limited quantity of primary studies, weak research designs and lack of accuracy in the PA and SES assessment methods employed. Further PA domain specific studies using longitudinal design and clear measures of SES and PA assessment are required.

## Background

The benefits of being physically active are well acknowledged in the primary and secondary prevention of many conditions such as cardiovascular disease, hypertension, type 2 diabetes, obesity, osteoporosis, anxiety and depression [1]. Compared to individuals who engage in regular moderate-intense physical activity (PA) for at least 150 minutes per week or 75 minutes per week of vigorous intensity, insufficiently physically active individuals have a 20–30% increased risk of all-cause mortality [1]. Evidence gathered from longitudinal studies undertaken in western countries show age-related changes in PA behaviours; with a steep decrease in PA levels occurring during adolescence [2,3]. Considering that PA behaviours established in youth tend to track into adulthood, PA promotion in youth has been deemed a priority in order to facilitate a carryover of active lifestyles into adulthood and to warrant a lifelong protection from other risk factors [4]. Cross-sectional global estimates of PA report 25% of adults between 18–65 years, 55% of older adults (> 65 years) and 81% of school-aged youth (11–17 years) are insufficiently active [2]. Therefore, it is crucial to identify factors having a potential effect on PA behaviours.

To establish experimental evidence related to PA behaviours’ determinants, there is a need of a clear understanding of associations or predictive relationships between variables [5]. In general, the term “determinant” is used to address causal variables, including correlates (i.e., multiple variables intervening in cause-effect relationships), mediators (i.e., variables influencing in a cause-effect relationship between variables), moderators (i.e., variables effecting the strength of a relationship between variables), and/or confounders (i.e., variables associated with the outcome distorting the observed relationships) [6]. Furthermore, definitions of PA also present terms that lead to inconsistencies resulting in different interpretations and outcomes [5]. In fact, PA differs in term of typology (i.e. unstructured daily activities, such as occupational PA (OPA) and leisure time PA (LTPA) and structured PA such as physical exercise, grassroots sports, and competitive sports, frequency (e.g. daily, weekly, monthly), duration (e.g. activity/rest patterns), and intensity (e.g. low, moderate, moderate-vigorous, vigorous, maximal efforts). Recently, the strong links between health enhancing PA, grassroots sports, and competitive sports for the development, transfer and/or implementation of active lifestyles have been recognised [7]. Therefore, when reviewing the literature on determinants of PA, a wide perspective should be considered.

Socioeconomic status (SES) or its derivatives (e.g. income, education and occupation) has been recognised as an important determinant of health and wellbeing because it influences people's attitudes, experiences and exposure to several health risk factors across the life course [8]. In particular, children who grow up in lower SES households have a higher risk of unhealthier lifestyles, cardiovascular disease [9] and all-cause mortality [10,11] than children who live in higher SES households. Stringhini and colleagues [12] also report that the combination of potentially modifiable unhealthy behavioural factors such as physical inactivity, smoking and poor diet could explain between 12% to 54% of the SES differences in mortality; with the relationship between SES and smoking, alcohol consumption, and poor diet being more consistent than that between SES and PA [13]. This consistency is likely to be due to the multi-dimensional nature of PA and SES that pose considerable methodological measurement problems [13]. Few studies have attempted to categorise PA by domain (e.g. LTPA; occupational PA (OPA)) while at the same time ascertaining its association with SES across the life course. Researchers may need to focus on single components and use them as proxies for overall PA and SES, or use composite scores of SES (e.g., deprivation index or socioeconomic position) and PA (e.g. total PA) in order to reduce methodological inconsistencies. Furthermore, identification of barriers to PA related to SES factors, such as educational background, employment and/ or available income should play a key role in the development and implementation of future interventions and policy [9,10].

To date, the scientific literature on socioeconomic correlates and determinants of PA behaviours has been dispersed throughout a number of systematic reviews, often focusing on one factor (e.g. education or parental income) in one specific age group (e.g. pre-school children or adults). The purpose of the present study is to provide a comprehensive and systematic overview of the scientific literature from previously conducted research to assess the importance and strength of the evidence related to socioeconomic correlates and determinants of PA behaviours across the life course, through an umbrella SLR of systematic literature reviews (SLRs) and meta-analyses (MAs). The principle reason for choosing the umbrella review methodology is that it allows ready assessment of whether review authors addressing similar review questions independently observe similar results and arrive at generally similar conclusions. The aim of an umbrella review is not to repeat the searches, assessment of study eligibility, assessment of risk of bias or meta-analyses from the included reviews, but rather to provide an overall picture of findings for particular questions or phenomenon [14]. An umbrella review's most characteristic feature is that this type of evidence synthesis only considers for inclusion the highest level of evidence, namely other systematic reviews and meta-analyses. The wide picture obtainable from the conduct of an umbrella review is ideal to highlight whether the evidence base around a topic is consistent or contradictory, and to explore the reasons for the findings [15].

## Methods

The current research was developed within the Thematic Area 2 of the DEterminants of Diet and Physical ACtivity Knowledge Hub (DEDIPAC-KH). To systematise and update the current evidence-base on the determinants and correlates of PA behaviours across the life course, a common protocol for the DEDIPAC-HK umbrella SLR was developed and registered in PROSPERO (PROSPERO 2014: CRD42015010616) [16]. This manuscript is drafted following the PRISMA checklist [S1 Checklist].

### Search strategy and eligibility criteria

An online search was conducted using the following electronic databases; MEDLINE, ISI Web of Science, Scopus and SPORTDiscus. SLRs and MAs that focused on the association between any determinant of PA or exercise or sport as main outcomes were considered. The following exclusion was implemented: i) SLRs and MAs of intervention studies; ii) SLRs and MAs focusing on specific clinical population groups (e.g., chronic disease); and iii) umbrella SLRs on the same topic (e.g. reviews of SLRs or MAs of epidemiological studies on variables associated with PA). The search was limited to publications in English during the period from January 2004 to September 2017. Table 1 shows the MEDLINE search strategy that was also used as template for the search strategies in the other databases. Throughout this work, the term determinant will be utilised to address any variable affecting PA independently from their role; whereas the term PA will include non-structured and structured activities independently from their frequency, duration, and intensity.

**Table 1:** Search strategy: Key words used for the literature search.

Set	Search terms
#1	“physical activit*” OR “physical exercise*” OR sport OR “motor activit*” OR “locomotor activit*” OR athletic* OR fitness OR “physical movement*” OR “physical performance*” OR “aerobic exercise*” OR “physical effort*” OR “physical exertion*”
#2	determinant OR determinants OR correlator OR correlators OR mediator OR mediators OR moderator OR moderators OR contributor OR contributors OR factor OR factors OR association OR modifier OR modifiers OR confounder OR confounders OR pattern OR patterns OR predictor*
#3	demographic* OR motivation OR cognition OR emotion* OR attitude* OR “self-perception” OR “self-confidence” OR “self-efficacy” OR competence OR reward* OR success* OR challenge* OR knowledge OR belief* OR “personal trait*” OR “body image” OR satisfaction OR “time availability” OR “perceived environment” OR family OR peer* OR school* OR leader* OR coach* OR group* OR “climate” OR network* OR employment OR retirement OR “educational level” OR SES OR “socioeconomic status” OR “local identity” OR “national identity” OR value* OR tradition* OR “social expectation*” OR “social trend*” OR “social barrier*” OR “availability of tool*” OR “availability of service*” OR “access to tool*” OR “access to service*” OR neighborhood OR “community route*” OR “school environment” OR “work environment” OR architecture OR urbanization OR transport OR traffic OR “facilit* in public space*” OR advertisement OR “availability of sport club*” OR “availability of fitness center*” OR advocacy OR lobbying OR “corporate social responsibility” OR “physical activity promotion initiative*” OR legislation OR health OR education OR tourism OR environment OR “urban planning” OR transport* OR sport OR sports OR culture OR dance OR theater OR “gender mainstreaming” OR “social inclusion” OR “fiscal measure*” OR program* OR plan OR plans OR communication OR media OR guideline*
#4	“systematic literature review” OR “meta-analysis”

### Selection process

The selection process consisted of three phases. In the initial phase, relevant articles were independently screened and assessed by two reviewers belonging to the DEDIPAC KH, who screened the yielded articles based on title. In the case of doubt, the articles were included in the abstract review phase. In the second phase, all articles selected from the initial phase had their abstract reviewed and assessed by two independent reviewers of the DEDIPAC- Knowledge Hub (KH) research team (KA and AP). Any uncertainty and disagreement was resolved by consulting three further authors (SB, LC, AP). In the final phase, AK and GO’D fully reviewed the remaining articles. In this phase, any disagreement between reviewers was resolved by discussion within the DEDIPAC-KH research team. In considering the specific focus of the present umbrella SLR, studies that focused on non-socioeconomic determinants of PA behaviours were not considered.

### Data extraction

A fourteen item standardised pre-piloted data extraction form was used to extract data from the included studies under the following headings: year of publication, type of review (either SLRs or MAs), number of eligible primary studies included in the represented umbrella SLR over the total number of studies included in the review; continent/s of the included studies, primary study design, overall sample size, age range or mean age, gender proportion, year range of included studies; outcome details, type of determinant/correlate, aim of the review; overall results (qualitative or quantitative), overall recommendations and limitations as provided by the review itself. Furthermore, the importance and strength of evidence of a determinant included in a particular review was evaluated by applying a modified version of the criteria adopted by the World Cancer Research Fund [17], further adapted by Sleddens et al [18].

### Outcome measures

The socioeconomic factors used to classify socioeconomic status in this review were based on those used by Beenackers et al (2012) in their review on socioeconomic inequalities in physical activity among European adults. The factors considered were income, referring to an individual’s or household income. Education, referring to the highest attained level of education (e.g. university education). Occupation, such as blue or white collar workers and other social economic predictor indicators such as home ownership and the ability to pay fees and / or purchase equipment required to engage in structured physical activity [19].

In terms of PA categories, total PA, moderate-vigorous PA (MVPA), LTPA and OPA were categorised where possible. In relation to age groups, the categories included: pre-school children (2–5 years), children (6–12 years), adolescents (13–18 years), adults (19–65 years) and older adults (> 65 years). Furthermore, break-time/recess time PA and after-school PA were also considered for pre-school children and children, while uptake of and adherence to exercise referral schemes were considered for adults.

### Risk of bias assessment

Assessing risk of bias of the reviews is essential because it impacts on the extent to which conclusions can be drawn from the evidence. A modified AMSTAR checklist [20] was used to perform the quality assessment of the included reviews. Two reviewers belonging to the DEDIPAC KH independently evaluated the included reviews. Any uncertainty and disagreement was resolved by consulting three further authors (SB, LC, AP). The eleven criteria were evaluated and scored with 1 when the criterion was met or with 0 when the criterion was not met.

As a consequence, the total quality score for each included SLR ranged from 0 to 11 quality scores. The quality of the SLR was labelled as weak (score range: 0–3), moderate (score range: 4–7), or strong (score range: 8–11).

### Data synthesis

A narrative synthesis of the findings of this umbrella SLR is provided, structured around a modified version of the criteria for grading evidence and the data extraction employed by Sleddens and colleagues [18,21]. Results retrieved from the eligible primary studies included in the reviews were summarised combining two grading scales. The first, grades the importance of the determinants, referring to the consistency of the associations among the reviews, or the individual primary studies. The second, grades the strength of evidence, referring to the study design used among individual primary studies.

According to Sleddens [18, 21], the codes + and ++ were used if there is an association (no matter of positive or negative). This was modified for the present review to report both the association and the direction of the association (Table 2). The importance was scored (—) if all reviews, without exception, found a negative association between the determinant and the outcome. A (-) score was given if the negative association was found in 75% of the included reviews or of the original primary studies. The importance of the determinant was scored a (0) if the results were mixed, or more specifically, if the variable was found to be a determinant and/or reported an association (either positive or negative) in less than 75% of available reviews or of the primary studies of these reviews. The importance of a determinant was scored as (00) if all reviews, without exception, reported a null association. The importance of the determinant scored (+) if a positive association was found in 75% of the reviews or of the included primary studies and (++), if a positive association was found in all reviews, without exception.

**Table 2:** Importance of a determinant.

Category of importance	Definition
**+ +**	The variable has been found to be positively associated in all reviews, without exception. This could mean that only one review has included a particular variable, and showed that this was a significant positive correlate and/or reported a (non)-significant effect size larger than 0.30, but it could also mean that a number of reviews were conducted that included this variable and all of them concluded that the variable was significantly positively related to the particular behavioral outcome.
**+**	This implies that > 75% of the available reviews concluded the variable to be positively related, or the separate reviews report that 75% or more of the original studies concluded the factor to be positively related. This could therefore mean that only one review has included a particular variable, and showed that this was a significant positive correlate in > 75% of studies. But it could also mean that a number of reviews were executed towards this variable and most, but not all, concluded that the variable was positively related to the particular behavioral outcome.
**0**	The variable has been found to be related and/or reported a (non)-significant effect size larger than 0.30 in some reviews (25% to 75% of available reviews or of the studies reviewed in these reviews), but not in others. The direction of the relationship can be either positive or negative. This could mean that only one review has included a particular variable, and showed ‘mixed findings’, but it could also mean that results are mixed across reviews.
**00**	The variable was found to have no association. It scored (00) if all reviews, without exception reported a null hypothesis
**-**	This implies that > 75% of the available reviews concluded the variable to be negatively related, or the separate reviews report that 75% or more of the original studies concluded the factor to be negatively related. This could therefore mean that only one review has included a particular variable, and showed that this was a significant negative correlate in > 75% of studies. But it could also mean that a number of reviews were executed towards this variable and most, but not all, concluded that the variable was negatively related to the particular behavioral outcome.
**- -**	The variable has been found to be negatively associated in all reviews, without exception. This could mean that only one review has included a particular variable, and showed that this was a significant negative correlate and/or reported a (non)-significant effect size larger than 0.30, but it could also mean that a number of reviews were conducted that included this variable and all of them concluded that the variable was negatively related to the particular behavioral outcome.

The strength of the evidence was also summarized using the criteria adopted by Sleddens et al [18,21]. The strength of evidence was described as ‘convincing’ (Ce) if it was based on a substantial number of longitudinal observational studies, with sufficient size and duration, and showing consistent associations between the determinant and PA. The strength of the evidence was defined as ‘probable’ (Pe) if it was based on at least two cohort studies or five cross control studies showing fairly consistent associations between the determinant and PA. The strength of the evidence was given as ‘limited, suggestive evidence’ (Ls) if it was based mainly on findings from cross-sectional studies showing fairly consistent associations between the determinant and PA, and as ‘limited, non- conclusive evidence’ (Lnc) if study findings were suggestive, but insufficient to provide an association between the determinant and PA.

The strength of the evidence (Table 3) was described as “convincing” if based on high quality studies showing consistent associations and having longitudinal design with sufficient size and duration, whereas “probable” strength of evidence was given to determinants showing fairly consistent associations based upon at least one cohort study. In the second case, shortcomings were possible either in terms of the consistency of the results or other aspects such as limited duration of the studies, small sample sizes or inadequate follow up. Furthermore, “limited suggestive evidence” was given to determinants for which there was insufficient number of longitudinal studies and “limited, no conclusive” evidence when the evidence for the associations between a determinant and the outcome were based solely on studies of cross-sectional design.

**Table 3:** Criteria for grading evidence.

Strength of evidence	Grading Criteria
**Convincing evidence (Ce)**	Evidence based on studies of determinants showing consistent associations between the variable and the behavioral outcome. The available evidence is based on a substantial number of studies including longitudinal observational studies and where relevant, experimental studies of sufficient size, duration and quality showing consistent effects. Specifically, the grading criteria include evidence from more than one study type and evidence from at least two independent cohort studies should be available, and strong and plausible experimental evidence.
**Probable evidence (Pe)**	Evidence based on studies of determinants showing fairly consistent associations between the variable and the behavioral outcome, but there are shortcomings in the available evidence or some evidence to the contrary, which precludes a more definite judgment. Shortcomings in the evidence may be any of the following: insufficient duration of studies, insufficient studies available (but evidence from at least two independent cohort studies or five case-control studies should be available), inadequate sample sizes, incomplete follow-up.
**Limited, suggestive** **evidence (Ls)**	Evidence based mainly on findings from cross sectional studies. Insufficient longitudinal observational studies or experimental studies are available or results are inconsistent. More well-designed studies of determinants are required to support the tentative associations.
**Limited, no** **conclusive evidence (Lnc)**	Evidence based on findings of a few studies which are suggestive, but are insufficient to establish an association between the variable and the behavioral outcome. No evidence is available from longitudinal observational or experimental studies. More well-designed studies of determinants are required to support the tentative associations.

## Results

**Fig 1:**
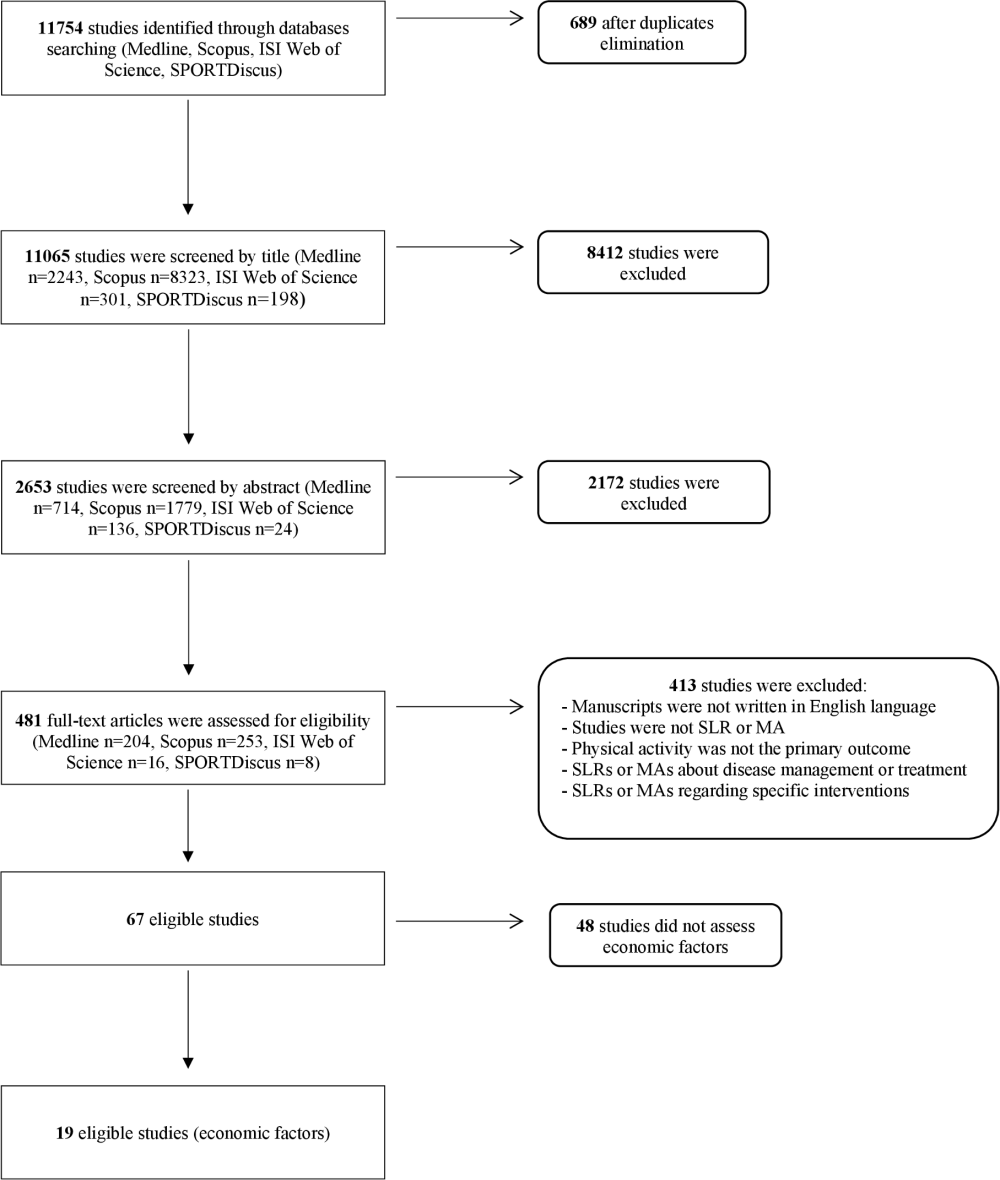
Flowchart of the literature research by database.

The process for undergoing the literature search and screening, including numbers of reviews excluded and reasons for exclusion is illustrated in Fig 1. In summary, the electronic search yielded 11754 records, of which 689 duplicates were removed. Of the remaining 11065 records, 10998 were excluded throughout the screening process. After the full-text reading phase, the final number of studies eligible for the umbrella review was 67. Of these, 48 did not concern socioeconomic factors. Therefore, the final number of reviews included in this umbrella SLR on socioeconomic determinants of PA was 19 SLRs. No MAs were found to be eligible.

### Review characteristics

Table 4 provides detailed characteristics of the 19 systematic reviews. Of the 19 included, only two reported socioeconomic factors in all their primary studies (n = 164) [19,22]. Most of the reviews included primary studies from multiple continents, except two reviews; one that considered only cohort studies conducted in Europe [19] and the other only Chinese cohorts [23]. In general, the majority of primary studies resulted from Europe (n = 206), followed by North America, (n = 96) and Oceania (n = 14). Cross-sectional (n = 117) was the predominant study design among the 19 SLRs, though eight reported longitudinal (prospective) studies. One SLR solely focused exclusively on qualitative studies [24], whereas another one used mixed (e.g. qualitative and cross sectional) methodologies [25]. In terms of sample size, a considerable variation (from 25 to 29135 participants) emerged. Regrettably, it was not possible to retrieve the total population sample size from one SLR [26].

**Table 4:** Characteristics of the included reviews.

Author, Date (Type of review) [Ref]	Number of eligible studies included in the umbrella review / total number of studies included in the review	Continent/s of included studies	Study design of included studies	Total sample size of included study (Sample range)	Age range or mean (years) of eligible studies	Gender (female, % range) of eligible studies	Year range of included studies
Barnett et al, 2012 (SLR) [38]	4/19	North America (n = 2) Europe (n = 2)	Longitudinal (n = 2), Cross sectional (n = 2)	699–11489	60–64 years	27–53	2005–2009
Beenackers et al, 2012 (SLR) [19]	131	Europe (n = 131)	Designs not reported (n = 131)	224 to 60938	18–65 years	0–100	2000–2010
Beets et al, 2010 (SLR) [35]	4/80	North America (n = 4)	Cross-sectional (n = 3), Qualitative (n = 1)	25–789	< 18 years	0–100	1970–2008
Coble et al, 2006 (SLR) [39]	10/35	North America (n = 10)	Cross-sectional (n = 10)	127–2912	>18 years	N.A.	1990–2005
Craggs et al, 2011 (SLR) [26]	15/46	North America (n = 8), Europe (n = 5, Australia (n = 2)	Longitudinal (n = 15)	N/A	4–18 years	N.A	Up to Nov 2010
De Craemer et al, 2012 (SLR) [27]	9/43	North America (n = 4), Australia (n = 3), Europe (n = 2)	Cross sectional (n = 8), Longitudinal (n = 1)	46–5852	4–6 years	N.A.	Jan 1990- Sept 2010
Edwardson et al, 2010 (SLR) [29]	1/96	North America (n = 1)	Cross-sectional (n = 1)	200–299	6-11years	0–100	Up to Sept 2009
Ferreira et al, 2006 (SLR) [33]	33/150	North America (n = 23,) Europe (n = 6), Oceania (n = 4)	Cross-sectional (n = 28), Longitudinal (n = 5)	100–4999	3–18 years	N.A.	1980–2005
Gidlow et al, 2006 (SLR) [22]	33/33	North America (n = 19), Europe (n = 10), Oceania (n = 4)	Cross-sectional (n = 29), Longitudinal (n = 4)	220085 (n = 84–61239)	16–70+ years	0–100	Up to Oct 2004
Gustafson et al, 2006 (SLR) [34]	6/34	North America (n = 4), Europe (n = 2)	Cross-sectional (n = 5), Longitudinal (n = 1)	59–3254	3–18 years	43–100	1985–1997
Hinkley et al, 2008 (SLR) [28]	4/24	North America (n = 2), Europe (n = 2)	Cross-sectional (n = 3), Longitudinal (n = 1)	33–339	3–5 years	N.A.	1980–2007
Lu et al, 2017 (SLR) [23]	9/42	China (n = 7)	Cross-sectional (n = 6), Longitudinal (n = 1)	50–29139	3–18 years	N.A.	2002–2016
Olsen et al, 2013 (SLR) [25]	14/21	North America (n = 14)	Cross-sectional (n = 5), mixed methods (n = 2), qualitative (n = 7)	5789 (n = 2–2338)	>18 years	100	N.A.
Pavey et al, 2012 (SLR) [37]	1/20	UK (n = 1)	Cross-sectional (n = 1)	3568	9–92 years	61.1	Up to Oct 2009
Ridgers et al, 2012 (SLR) [30]	3/53	Australia (n = 1), Asia (n = 2)	Cross sectional (n = 3)	3406 (n = 80–2946)	4–13	32.5–56	Jan 1990- Apr 2011
Siddiq et al, 2011 (SLR) [24]	8/29	North America (n = 8)	Qualitative (n = 8)	414 (n = 19–92)	19–85	48.3–100	Up to May 2010
Stanley et al, 2012 (SLR) [32]	2/22	North America (n = 2)	Cross sectional (n = 2)	9194 (n = 1556–7638)	10–15	52.2–100	1999–2010
Van der Horst et al, 2007 (SLR) [31]	8/57	North America (n = 5), Australia (n = 1), Europe (n = 2)	Cross sectional (n = 8)	49480 (n = 87–17766)	11–24	47–100	1999–2004
Wendel-Vos et al, 2007 (SLR) [36]	6/47	North America (n = 4), Australia (n = 2)	Cross-sectional (n = 6)	48268 (n = 350–29135)	>18 years	N.A.	1980–2004

Eleven SLRs considered primary studies that included only the young population, with pre-school children being the focus of two SLRs [27,28], children of two [29,30], adolescents of two [31,32], and a combination of children and adolescents of five [23,26,33,34,35]. Eight SLRs focused only on adults [19,22,24,25,36,37,38,39], with two also considering older adults [24,37].

### Quality assessment

Table 5 summarises the quality assessment of the19 SLRs. The majority (n = 15) of the SLRs showed a moderate quality (range: 4–7 points), three [19,31,35] were evaluated as weak (range: 0–3 points) and only one [37] as strong (8-11pts).

**Table 5:** Quality assessment of the included reviews using the AMSTAR checklist.

Author, Date [Ref]	Was an 'a priori' design provided?	Was there duplicate study selection and data extraction?	Was a comprehensive literature search performed?	Was the status of publication (i.e. grey literature) used as an inclusion criterion?	Was a list of studies (included and excluded) provided?	Were the characteristics of the included studies provided?	Was the scientific quality of the included studies assessed and documented?	Was the scientific quality of the included studies used appropriately in formulating conclusions?	Were the methods used to combine the findings of studies appropriate?	Was the likelihood of publication bias assessed?	Was the conflict of interest included?	Sum quality score	Quality of the review
**Barnett, 2012 [38]**	No	Yes	Yes	Yes	No	Yes	Yes	Yes	Yes	C.A.	No	7	Moderate
**Beenackers, 2012 [19]**	Yes	No	C.A.	No	No	Yes	C.A.	No	N.A.	No	No	2	Weak
**Beets, 2010 [35]**	Yes	C.A.	Yes	N.A.	No	No	No	C.A.	Yes	N.A.	C.A.	3	Weak
**Coble, 2006 [39]**	No	No	Yes	Yes	No	Yes	No	No	Yes	No	No	4	Moderate
**Craggs, 2011 [26]**	Yes	Yes	No	No	No	Yes	Yes	Yes	N.A.	No	Yes	6	Moderate
**De Craemer2012 [27]**	Yes	Yes	Yes	No	No	No	No	N.A.	N.A.	No	Yes	4	Moderate
**Edwardson, 2010 [29]**	Yes	C.A.	Yes	N.A.	No	No	No	Yes	Yes	N.A.	C.A.	4	Moderate
**Ferreira, 2006 [33]**	Yes	No	Yes	Yes	No	Yes	No	No	No	No	Yes	5	Moderate
**Gidlow, 2006 [22]**	Yes	C.A.	Yes	Yes	No	Yes	Yes	Yes	Yes	N.A.	No	7	Moderate
**Gustafson, 2006 [34]**	Yes	C.A.	Yes	N.A.	No	Yes	No	Yes	Yes	N.A.	Yes	6	Moderate
**Hinkley, 2008 [28]**	Yes	Yes	Yes	N.A.	No	No	No	No	N.A.	No	Yes	4	Moderate
**Lu, 2016 [23]**	Yes	Yes	Yes	N.A.	No	Yes	No	No	Yes	No	Yes	7	Moderate
**Olsen, 2013 [25]**	Yes	No	Yes	No	No	Yes	Yes	No	N.A.	No	C.A.	4	Moderate
**Pavey, 2012 [37]**	Yes	Yes	Yes	No	Yes	No	Yes	Yes	Yes	Yes	Yes	9	Strong
**Ridgers, 2012 [30]**	Yes	C.A	Yes	No	No	Yes	No	N.A.	N.A.	N.A.	Yes	4	Moderate
**Siddiq, 2011 [24]**	Yes	No	Yes	No	No	Yes	Yes	Yes	N.A.	No	Yes	6	Moderate
**Stanley, 2012 [232]**	No	Yes	No	No	No	No	Yes	Yes	N.A.	No	Yes	4	Moderate
**Van der Horst, 2007 [31]**	No	Yes	Yes	No	No	Yes	No	N.A.	N.A.	No	No	3	Weak
**Wendel-Vos, 2007 [36]**	No	Yes	Yes	No	No	Yes	No	No	No	No	Yes	4	Moderate

C.A. Can’t answer N.A. not applicable

### Major findings

Table 6 summarises the findings of the SLRs on the associations between the socioeconomic determinants and PA. The most frequently studied correlates were SES (n = 12) [19,22,23,26,27,28,30,31,32,33,34,38], payment of fees or equipment (n = 5) [24,25,29,35,36], education level (n = 6) [22,23,25,28,33,39], and individual or household income level (n = 6) [22,25,33,36,37,39]. In addition, neighbourhood income (n = 2) [36,37], employment levels (n = 2) [36,37] and number of working hours of an individual or parent (n = 2) [25,33] were also examined. Finally, two SLRs [33,33] also considered parental occupational status and home ownership determinants of PA in children. In combining the resulting importance of the correlates or determinants (e.g., the number of included primary studies or the number of SLRs that showed an association between socioeconomic determinant and PA and the strength of evidence (e.g., the study design of the primary studies) [17]. Table 7 summarises the final judgments on the associations between the investigated socioeconomic determinants and PA. The following paragraphs refer to age-related findings for individuals <18 years (e.g., children and adolescents) and >18 years of age (e.g., adults).

**Table 6:** Results of the included reviews.

Author, Date [Ref]	Outcome(s)	Determinant(s)	Review aim	Overall qualitative results of the review	Overall quantitative results of the review	Overall limitations of the study	Overall Recommendations
**Barnett et al, 2012 [38]**	(i) Changes in overall physical activity, (ii) changes in leisure-time PA (incl. Exercise but also hobbies and other active recreation	Retirement transition (SES influence on PA during the retirement transition). SES was determined by occupation in all four studies. Two of the studies also used household wealth as a SES indicator.	To examine changes in physical activity across the retirement transition, whether these changes vary by SES and what is known about predictors of these changes	Moderate evidence that retirement from manual or low-grade occupations was consistently associated with lower PA after retirement. Three studies reported a positive association between PA and retirement from sedentary or high grade occupation.	NA	Only 4 studies and only looking at SES influence on changes of PA due to retirement	SES moderates the association; lower SES is consistently associated with decreased PA levels and higher SES with increased PA levels. Findings should be interpreted with caution because robust assessment of SES can be challenging in retirement. For example, the use of last occupation to determine SES might lead to misclassification because of the downward mobility experienced by many employees in later working life.
**Beenackers et al, 2012 [19]**	Total PA, total LTPA, (LTPA was the most common measure (n = 112)) active transport and OPA	Indicators of SEP included education, social class, (based on occupation), income (either individual or household level), household wealth (car, home ownership) or area-based indicators (area deprivation)	To systematically review the evidence pertaining to socioeconomic inequalities in different domains of PA by European region	Considerable differences in the direction of inequalities were seen based on different domains of PA. Most studies reported those with a high socioeconomic position were more physically active during leisure-time compared to those with lower socioeconomic positioning (68% +ve association for total LTPA) Occupational PA was more prevalent among lower SE groups (63% negative associations). Socioeconomic differences in total PA and active transport PA did not show a consistent pattern.	NA	English language only, methodological differences such as PA assessment, participant selection and the adjustment for confounders in some studies	LTPA and more specifically, vigorous LTPA is less prevalent while occupational PA is more prevalent among people with lower SEP. Inconsistent results in total PA indicate that total PA may not be a suitable summary measure when investigating inequalities in PA.
**Beets et al, 2010 [35]**	Physical activity was defined as any bodily movement that results in energy expenditure. This definition allowed for the inclusion of both organised (e.g. sports) and non-organised (e.g. free play) physical activities	Payment of fees and / or equipment	To systematically examine the relationship of parental social support to physical activity related behaviours of youth.	Only four studies (6%) assessed the association of parental payment of fees and purchase of equipment to activity-related behaviours. Though limited in numbers, these studies indicate that parents purchase more equipment for boys than girls and payment of fees has been associated with higher levels of activity. High costs associated with involvement are rated as the number one reason for nonparticipation in school-community sports and non-sport activities.	NA	Only 4 studies examining the association of parental payment of fees and purchase of equipment	Variations in whether or not parental social support is related to youth activity levels is shown to be a function of the activity measure employed; Inconsistent measurement of physical activity needs to be addressed. Future studies should also consider the effect of negative family support on physical activity participation, one of the studies reported this to be inversely related to boy's PA levels
**Coble et al, 2006 [39]**	Physical activity was defined as any bodily movement that results in energy expenditure. Therefore, any study that measured leisure-time PA, occupational activities, household activities, exercise and sport was included	Education, employment and income	To unite the literature regarding the physical activity behaviours of Native Americans	Education was not related to PA in 3 of the 5 studies. In the remaining two, it was shown that women with a college education were three times more likely to participate in PA. The three studies that investigated employment found no relationship to PA levels and one of the 2 studies that investigated income found mixed positive associations between PA and income.	NA	Appropriateness of the measurement tools for PA; not validated for a Native American Population	This study was focused on Native Americans _ interestingly the findings are inverse in relation to PA and correlations with income, education and employment when compared to white Americans and Europeans
**Craggs et al, 2011 [26]**	Overall PA	Socioeconomic status	To systematically review the published evidence regarding determinants of change in PA in children and adolescents	Results were mixed: *< 9 years*: 2 studies were positively associated, 1 negatively and 3 studies report no relationship *10–13 years*: 3 studies were positively associated, 2 negatively and 6 studies report no relationship *>14years*: 1 study was positively associated and 2 showed no relationship	N.A.	Heterogenous studies in terms of study design, analysis methods, outcome measures and investigated exposures.	
**De Craemer et al, 2012 [27]**	Total physical activity and MVPA	Socioeconomic status	To review the correlates of physical activity, sedentary behaviour and eating behaviour in children between 4 and 6 years old	No association between SES and PA were identified	NA		
**Edwardson et al, 2010 [29]**	Overall PA levels and change in PA levels over time	Payments of fees	To examine parental influences on different types and intensities of PA in youth	For boys parental fee paying significantly related to PA and to PA changes. There was no relationship evident for girls	NA	Only one study investigated the variable (payment of fees)	
**Ferreira et al, 2006 [33]**	Any measure of overall PA of various types (i.e. play, games, sports, work, transportation, PE or planned exercise) expressed in duration or frequency or a combination of both (METS or kcal)	Parental SES, Parental occupational status, father occupational status, mother occupational status, parental education, father's education, mother's education, hours parents work, home ownership	To systematically review the environmental factors that are associated with PA in youths	Parental SES was positively associated with youth PA in 8 studies, negatively associated in 1 and not related in 13. Parental occupational was examined in 6 studies and found not be related to youth PA levels. Parental education was not related in 21 out of 24 studies. No relationship with PA levels and home ownership (n = 2) was identified	NA	Heterogeneity of PA measures and weak study designs	Studies in which parental SES was defined as a composite of parent's education and income levels / occupational status were generally unrelated to children's PA. However, studies in which the individual component of SES were examined showed some positive association
**Gidlow et al, 2006 [22]**	Overall PA, LTPA, VPA, MPA and MVPA, walking, habitual activity, household activity, change in PA	Social Class, Income, Education and Home ownership	To determine if there is strong evidence of a positive gradient increasing physical activity across the socioeconomic strata and how relationships are effected by socio-economic measurement	Consistent evidence of a higher prevalence or higher levels of LTPA or MVPA in those at the highest socioeconomic strata compared with those at the bottom. Evidence for positive gradients across the socioeconomic strata was less consistent. Education produced the most stable relationship, less susceptible to confounding effects of ethnicity / environment.	NA	The majority of studies were secondary analyses of existing health survey data, which could explain the generally large sample sizes and methodological weaknesses in physical activity and SEP measurement.	Diverse and often crude physical activity and socio-economic measurement made it difficult to distinguish and true effect with so many potential confounding influences. Further studies using contemporary methods of socio-economic and physical activity measurement are necessary to further explore this relationship and its confounders
**Gustafson et al, 2006 [34]**	Physical activity was defined as any bodily movement that results in energy expenditure. This definition allows for the inclusion of both organised (e.g. sports) and non-organised (e.g. free play) physical activities	Family socioeconomic status	To unite the existing research on parental influences on children's PA behaviours in order to establish direction for future research and improve existing PA intervention programmes	There was no significant relationship identified in any of the studies in terms of PA levels and family SES. One study showed that girls aged 9–12 were more likely to stay active as they got older if they were from a higher SES.	NA	80% of parents in two of the included studies had a minimum of a college education that may have influenced the study outcome. Also a major limitation is the lack of standard measurement of family SES. Confounders such as ethnicity and parental occupation were not controlled for	
**Hinkley et al, 2008 [28]**	All types of overall PA domains were included	SES, parental education	To systematically review articles investigating the correlates of preschool children's physical activity.	SES was investigated in 3 studies and found to be unrelated to PA levels in all three (0) and parental education was investigated in one study and was also not related to PA (0)	NA	The majority of research conducted in this area has employed relatively small potentially non-representative samples (usually with <300 participants).	The level of variability in PA is relatively small in preschool children thereby compounding the effect of small sample sizes
**Lu et al, 2016** **[23]**	Total PA or leisure time PA (LTPA)	Family SES, parental education	To systematically review the factors relating to physical activity in Chinese children and adolescents	There was no significant relationship identified in any of the studies in terms of PA levels and family SES or parental education levels	NA	Weak study designs (cross-sectional) and poor validated PA measurement tools	Future research is required to enhance understanding of influences, such as SES especially in early childhood.
**Olsen et al, 2013 [25]**	Overall PA levels	Work hours and demands, income, higher education, Lack of Access (transportation difficulties, lack of resources, lack of affordability)	To investigate the determinants of PA levels among rural women in the United States	Work hours and demands were identified as a barrier to PA, creating a need for adaptation. Income: 2 studies noted a correlation between increased income and increased levels of PA. Other showed income as an influence on other determinants e.g. transportation problems and an inability to afford fitness fees.	NA	Self-reported only PA was used; exclusion of articles outside US and only one reviewer was involved	Rural women have been found to be less active and experience more barriers to PA than urban women. Interventions to promote PA in rural women should address each of these dimensions (personal, socio-economic and physical) for optimal effectiveness
**Pavey et al, 2012 [37]**	Exercise referral scheme (ERS) uptake or adherence	Deprived neighbourhoods	To quantify the levels of ERS uptake and adherence and to identify factors predictive of uptake and adherence; to also identify differences in uptake and adherence between those recruited into observational studies and RCTs	Participants residing in more deprived neighbourhoods were less likely to attend at least one session and those individuals living in a more rural location where less likely to uptake ERS.	NA	Difficulty with the definitions of uptake and adherence	
**Ridgers et al, 2012 [30]**	Physical activity levels during school recess	Socioeconomic status	To examine correlates of children’s and adolescents’ physical activity during school recess periods	There was inconclusive evidence for associations between socio-economic status and physical activity. Some positive associations were found between overall facility provisions, unfixed equipment, and perceived encouragement and recess physical activity. Results also revealed that boys were more active than girls.	NA	Small sample sizes that utilised cross-sectional study designs. Limited number of studies that report effect sizes and the lack of consistency in the correlates assessed between studies. While the majority of child studies have used objective measures of physical activity, in particular accelerometry and direct observation, different accelerometer cut-points and observation systems may have influenced the strength of the associations observed.	There is currently a dearth of literature concerning correlates of physical activity during recess periods, particularly in adolescents. Despite the paucity of associations identified, it is recommended that schools should increase overall facility provision, provide unfixed equipment, and identify methods to increase social support, particularly by peers, to benefit children and adolescents’ physical activity levels during recess
**Siddiq Z, 2011 [24]**	Overall PA participation	Monetary/socio-economic issues	Aims to systematically examine and summarise factors impacting physical activity participation among African American adults	Monetary costs of joining a fitness club or purchasing exercise equipment were perceived as impediments, mostly among women	NA	The study does not aim to assess cross-cultural differences pertaining to perceptions of physical activity between various racial/ethnic groups, an area of investigation that clearly warrants further examination. Qualitative research has its limitations, such as inability to generalise findings to a reference population.	To effectively promote physical activity among African Americans, targeted interventions will need to address impediments at multiple levels: i.e. individual impediments.g. self-efficacy, lack of time and money), social barriers (e.g. lack of childcare and social support) and environmental determinants (e.g. crime in and lack of access to safe exercise facilities and parks).
**Stanley AM, 2012 [32]**	Break time and after-school PA.	Family affluence (SES)	To assess potential correlates of school break time and after-school PA	Positive associations were found for SES (family affluence) and school break time PA. No associations were found for SES and after school PA (females only study)	NA	The current review identified a small number of studies that varied widely in important methodological aspects, thereby limiting the generalisability conclusions that can be drawn. Due to the relatively high proportion of cross-sectional studies included in the review, it is not possible to identify those correlates of PA behaviour change that would provide the most powerful evidence for intervention design. The relatively narrow age range specified in the current review is a limitation.	This review exposed a lack of clarity in this area and underscores the importance of focusing attention on context- and setting-specific PA among young people. Associations between PA and a potential correlate have been shown to differ depending on how PA or the correlate was measured. Future studies should choose measurement tools with appropriate psychometric properties.
**Van der Horst K, 2007 [31]**	Overall PA	Socioeconomic status	To summarise and update the literature on correlates of PA, insufficient PA, and sedentary behavior in young people	No association between socioeconomic status and overall PA was found.	NA	Publication bias may be present; possibility of missed studies as a result of the search strategy; the main outcome was overall PA without other classifications; mostly cross-sectional studies included; because of the variability, it was not possible to assess the overall strength of the associations	More prospective studies are needed and more research including children
**Wendel-vos, 2007 [36]**	Neighbourhood walking, sedentary lifestyle, bicycling, active commuting, overall PA, LTPA, VPA, MPA and MVPA	Cost of PA, house hold income; accessibility of employment; neighbourhood income	To gain insight into which environmental factors have been identified as potential determinants of various types and intensity of physical activity among adult men and women.	Availability of physical activity equipment was convincingly associated with vigorous physical activity/ sports and connectivity of trails with active commuting. Other possible, but less consistent correlates of physical activity were availability, accessibility and convenience of recreational facilities. No evidence was found for differences between men and women.	NA	Most studies used cross-sectional designs and non-validated measures of environments and/or behaviour	The vast majority of environmental determinants included in this review resulted in null associations, implicating that there either is no association for these attributes or they were defined in a wrong way. Therefore, it is important to conduct future research with clear, possibly standardised definitions of environmental attributes and physical activity within the strongest study design possible

**Table 7:** Summary of the results of the included reviews: The importance of a determinant and its strength of evidence.

	Preschool children (Overall PA)	Children & Adolescents (Overall PA)	Children (Overall PA)	Adolecents (Overall PA)	Children & Adolescents Break Time	Children & Adolescents After School	Adults (Overall PA)	Adults VPA	Adults MVPA	Adults LTPA	Adults >40 (Ex referral schemes adherence)	Adults >65 (overall PA and LTPA)	Rural women	Native American	African American
**Determinant**															
**SES (individual, parental or family)**	00, Pr [23,27,28]	0, Pr [23,30, 33, 34]	0, Pr [26,31]	0, Pr [23,26, 31, 34]	+, Lns [30,32]	0, Lns [32] Female only	+, Ce [19,22]			+, Ce [19,22, 38]		+, Ls [38, 22]			
**Education level (individual or parental)**	00, Lns [28]	00, Pr [23,33]	+, Lns [34]	00, Pr [23]			+, Pr [22]			+, Pr [22]			+, Lns [25]	0, Ls [39]	
**Income (individual, parental or household)**		+, Ls [33]					0, Pr [22, 36]	0, Lns [22, 36]	0, Lns [22]	+, Pr [22]			+, Lns [25]	0, Ls [39]	
**Neighbourhood Income**										00Lns [36]	00, Lns [37]				
**Employment**														00, Ls [39]	
**Parental occupation**		+, Pr [33,34]													
**Work Hours (individual or parental)**		00, Pr [33]											-, Lns [25]		
**Payment of fees/equipment**		0, Ls [35]	+, Lns [29]				+, Lns [36]	+, Lns [36]	+, Lns [36]	+, Lns [36]			-, Lns [25]		-, Lns [24]
**Home Ownership**		00, Ls [33]							+, Lns [22]	+, Lns [36]					

### Children and adolescents (3–18 years)

In preschool children, overall SES was consistently found to be unrelated to overall PA levels [23,27,28], with a probable level of evidence *(00*, *Pe*). Similarly, it was consistently found to be unrelated to moderate and vigorous activity levels (MVPA) [27] in more than 75% of the primary studies assessing pre-school children and this category of determinants, resulting in a probable level of evidence *(0*, *Pe)*.

Among reviews that combined children and adolescents, SES was also found not to be significantly related to overall PA levels [33,34], with 75% of the primary studies reporting no relationship to SES, thus the evidence was rated as probable *(0*, *Pe)*. However, in SLRs that addressed children and adolescents separately, between 25–75% of SLRs found some evidence (mixed findings) that SES influences overall PA levels. Considering that some reviews found SES to be a correlate of overall PA, while others did not [26,31,34], the evidence is *(0*, *Pe*).

In terms of the specific domains of PA and SES considered in this age group, results were mixed. Limited and inconclusive evidence *(+*, *Lns)* showed school break time PA to be associated with SES in children and adolescents [32]. In contrast, SES was not associated with after school PA in children and adolescent girls, but again the level of evidence was limited and not conclusive (*0*, *Lns*) [32].

In terms of the relationship between parental education level and PA in children and adolescents, <25% of the primary studies of the SLRs showed any significant association [34], with a probable level of evidence *(0*, *Pe)*. Similarly, among SLRs that included only children or pre-schoolers, no association was found between parental education level and overall PA; though the level of evidence was limited *(00*, *Lns)* due to a limited number of primary studies (n = 10), most of which were cross-sectional (n = 10) in design.

Parental income, another measure of SES [33] can be added to the list of the factors associated with overall PA for children and adolescents, however the evidence available is limited *(+*, *Ls)* while parental home ownership [33] was found to have no correlation with overall PA in the same group *(0*, *Ls)*. There were mixed results in relation to parental occupation and overall PA, with parental occupation found to be a determinant in some reviews (25% to 75% of available reviews or of the studies reviewed in these reviews), but not in others *(+*, *Pe)* [33,34]. However, number of parental working hours consistently showed no effect on overall PA levels with a probable level of evidence *(00*, *Pe)* [31]. Payment of fees or equipment required for PA [32] showed divergent results with limited suggestive evidence. A consistent association was found between payment of fees or equipment and overall PA in reviews combining children and adolescents *(+*, *Ls)*, while another SLR on children only [29] consistently found no association with payment of fees or equipment and overall PA; though evidence was limited and not conclusive [29].

### Adults (>18 years)

Among adults aged over 18 years, it emerged that SES [19,22,39] was the sole correlate, with convincing evidence in the majority of the available original studies *(+*, *Ce)*. In addition to total PA, LTPA *(+*, *Ce)* and OPA *(-*, *Ce)* [19] were also found to be associated with SES, with a convincing level of evidence (>75% of the available original studies). Similarly, among older adults (> 65 years old), SES [38] was associated with overall PA and LTPA *(+*, *Lns*), though the evidence was limited.

There were mixed results in relation to individual income and overall PA, with income found to be a determinant in some reviews (25% to 75% of available reviews or of the studies reviewed in these reviews), but not in others *(0*, *Pe)* [22,36]. Furthermore, there was limited non-conclusive evidence *(+*, *Lns)* that income was associated with moderate and vigorous PA, based mainly on findings from cross-sectional studies and mixed non-conclusive evidence *(0*, *Lns)*. There was limited non-conclusive evidence that neighbourhood income was not a correlate of leisure time PA *(00*, *Lns)*. Similarly, neighbourhood income was unrelated to adherence to exercise referral schemes in adults over 40 years of age *(00*, *Lns)* [37]. Yet, there was some evidence that neighbourhood income was a factor when it came to the uptake of exercise referral schemes *(+*, *Lns)* [37] but the level of evidence was limited due to the low sample size and cross-sectional design of studies.

Single SLRs considered sub-categories adults; with one particular category being rural women [25]. In this sub-group, education level, income, payment of fees and equipment costs showed a limited non-conclusive association (+*+*, *Lns*). Furthermore, limited suggestive evidence *(++*, *Ls)* emerged for the number of hours spent working. Another specific group investigated was Native Americans [39]. In this sub-group, employment was not correlated to overall PA (*00*, *Ls)*, whereas findings related to educational levels and income were mixed (*0*, *Ls)*. Finally, a qualitative SLR [24] considered the sub-group African-Americans. In this sub-group payment of fees and/or equipment costs were perceived as impediments to PA, mostly among women, though the level of evidence was limited and non-conclusive *(++*, *Lns)*.

## Discussion

This is the first umbrella SLR that provides a detailed overview of reviewed research regarding economic factors that influence PA across the life course. Factors studied most frequently among all age groups that demonstrated evidence of some association with PA, particularly in adults, were overall SES, income, payment of fees, and /equipment costs for PA. However, because of the general use of cross sectional designs in the studies covered in the available reviews, the evidence for true determinants is suggestive at best.

The included SLRs suggest that for adults, overall SES is the sole factor identified, with convincing evidence to be significantly related to overall PA, OPA and LTPA. This finding was also evident for older adults (>65 years), though the strength of the evidence was less convincing. In the reviews that examined specific components associated with SES such as education and income, the evidence was less consistent, resulting in mixed findings for overall PA and LTPA, with a probable level of evidence. The reasons behind these mixed results, as reported by the reviews are: small sample sizes, high diversity of the population included between studies and the diversity of the measurement methods of PA used among the primary studies.

While most associations were not statistically significant, both positive and negative associations were identified between educational level and PA with the direction of the relationship between education and PA being domain dependent. For example, one particular study found educational level to be positively associated with LTPA and negatively associated with OPA [19]. Similarly, in fact, the most recent data on PA from the European Commission [40] indicates that high socio-professional categories (e.g. managers, white collars, and self-employed) tend to engage in PA more frequently compared to the unemployed, retired, and those that work in the home. Furthermore, the majority (68%) of European citizens with a limited educational level (≤ 10 grade; 15yrs of age) report never exercising or playing sport, whereas those who ended education at 16–19 years and ≥20 years was 47% and 27%, respectively. These findings highlight the importance of domain specific PA research to accurately assess its association with different socioeconomic determinants. Distinguishing between PA behaviours by purpose (e.g. work, leisure), environment (e.g. location, type of community, physical environment), type (e.g. exercise) and time (e.g. time of the day, month or year) might assist researchers to further identify the determinants of specific PA behaviour and mitigate inconsistencies from previous studies.

Despite convincing evidence for the impact of SES measures on adult PA levels, the findings were less convincing for children and adolescents (< 18 years). There was no association with SES and overall PA in preschool children (3–6 years). In school-aged children and adolescents, (6–17 years), the findings were inconsistent. Socioeconomic indicators were not related to PA in children and adolescents when the groups were considered as one. When separating these groups, the findings varied, with some studies showing a relationship with SES and overall PA, while others showed no relationship. This may be due to the vastly different age ranges of children and adolescents (4–18 years) and a dramatic decline in PA from childhood to adolescence [41]. It is perhaps understandable that when youth is considered as one it is difficult to accurately identify associations.

Many changes with respect to physical development and social interactions are taking place during the transition from childhood to adolescence that directly influences PA levels. For example, when children are young, parents are highly responsible for their access to PA. However, parental influence decreases with advancing age, as the child gains more independence and is increasingly exposed to other environments (e.g. school environment, peer influences). Cost of PA has also been shown to be somewhat influenced dependent on the child’s age [41]. Among younger children, PA is most frequently informal in nature and scarcely gives rise to additional costs. In older children and adolescents, it often has more costs associated with it through membership of sports clubs and purchase of equipment, which can result in socially disadvantaged adolescents being less likely to remain, or become, active.

Another reason for the absence of a consistent direction in the SES inequalities and total PA in children and adolescents might be caused by the fact that PA is a complex variable with many components. One SLR looked at two specific activity domains for children and adolescents (e.g. break-time PA and after school PA) [32]. Stanley et al [32] showed that there was a consistent relationship between SES and break-time PA in both children and adolescents, while no consistent relationship between SES and after school PA was identified; albeit the level of evidence was weak and for females only. During the school day, physical education and playtime enables children to engage in regular PA, although it has shown these increases have only made a small contribution to total daily PA [30].

Based on the current literature, one may conclude that the importance of SES measures throughout the youth may increase with age but the sparse evidence needs further clarification. As with adults, investigating PA from a domain specific perspective in children and adolescents appears to be essential to achieve a detailed understanding of the determinants of SES measures on PA.

### Limitations of the study

The main limitation that we have identified with regards to this umbrella review is the lack of SLRs available for inclusion. Only two SLRs [19,22] looked at SES or its derivatives (income, education, employment) as their primary outcome. The remainder of the included SLRs focused primarily on social, biological and environmental determinants and included on average only two studies per SLR that investigated potential socioeconomic correlates. Furthermore, most of the individual studies looked at only one component of SES such as income, education, or payment of fees in relation to PA; making both quality and strength of evidence assessment difficult.

Certain general limitations and assumptions of SES studies should be considered. Firstly, SES is a theoretical construct involving various measures (e.g., income, occupation, education) that tap into different components of this construct [42]. However, there is no overarching agreement in the literature on the use of specific SES measures and SES definitions applied in the studies often differ. Thus, a reported association between a given SES measure and PA may not always be consistent with findings observed in other studies due to inconsistent SES definitions employed. Although SES is the measure most commonly used in the reviews examined, educational level, income and occupation were also looked at in some reviews.

Umbrella reviews can be prone to bias in various ways. The SLRs examined in this umbrella review had mostly primary studies of a cross-sectional design and their findings were limited, in that only association could be established. Therefore, it was impossible to truly identify determinants. Significant differences in reviewing methodology and reporting were apparent. Most of the included reviews were of moderate methodological quality; with only one review having strong methodological quality. The majority of the SLRs did not include grey literature and the probability of publication bias was rarely assessed. Additionally, 18 out of the 19 SLRs did not provide lists of excluded studies and most did not assess and document the scientific quality of the included studies. Finally, there were no existing approved criteria for grading the evidence of the individual SLRs included in the umbrella review. In order to try and increase the relevance and comparability of our results, we therefore used grading methods that were applied in previous reviews of this kind [17,18,19].

By their nature, umbrella reviews lead to loss of detail, with some individual studies included in multiple SLRs. This may have led to an overrepresentation of single studies in our results. The majority of the studies included in the SLRs were also conducted in the developed world (Europe, North America and Australia). As a consequence, some socioeconomic determinants that maybe more relevant in less developed countries, or countries with greater inequalities, may not have been identified.

## Conclusions

This is the first umbrella review providing an overview of socioeconomic correlates of PA across the life course. While some evidence is available for adults, less was available for youth. Specific SES measures identified such as overall SES, educational level, income and payment of fees/equipment for PA, should be used to develop and steer interventions and programmes in accordance with the target group. Individual and population-related interventions should primarily be targeted on correlates that can be strongly influenced and are most likely to bring about behaviour changes.

For future studies, it would be advisable not only to ensure an appropriate study design but also to use consistent, reliable and validated measurement methods in the assessment of PA and socioeconomic correlates. Using multiple domain measures of PA would also provide a more complete description of their associated correlates. An attempt to further understand the impact SES and its individual components has on adults, as well as on the parent-child PA relationship, is essential. Finally, in order to reach the most disadvantaged groups, a better understanding of the interrelationships between the dimensions of socio-economic circumstances and their effects on PA is paramount.

## Supporting information

S1 Checklist PRISMA checklist. (DOC)

## Abbreviations

DEDIPAC-KH: Determinants of Diet and Physical Activity–Knowledge Hub
PA: physical activity
SLR: systematic literature review
MA: meta-analysis
SES: socioeconomic status
LTPA: leisure time physical activity
OPA: occupational physical activity
MVPA: moderate to vigorous physical activity
Ce: convincing evidence
Lns: Limited, no conclusive evidence
Ls: Limited suggestive evidence
Pe: Probable evidence
